# A New NO-Releasing Nanoformulation for the Treatment of Pulmonary Arterial Hypertension

**DOI:** 10.1007/s12265-016-9684-2

**Published:** 2016-03-09

**Authors:** Nura A. Mohamed, Blerina Ahmetaj-Shala, Lucie Duluc, Louise S. Mackenzie, Nicholas S. Kirkby, Daniel M. Reed, Paul D. Lickiss, Robert P. Davies, Gemma R. Freeman, Beata Wojciak-Stothard, Adrian H. Chester, Ibrahim M. El-Sherbiny, Jane A. Mitchell, Magdi H. Yacoub

**Affiliations:** Department of Cardiothoracic Pharmacology, National Heart and Lung Institute, Imperial College, Dovehouse Street, London, SW3 6LY UK; Heart Science Centre, Imperial College, Harefield, Uxbridge, UB9 6JH UK; Qatar Foundation Research and Development Division, Doha, Qatar; Department of Experimental Medicine and Toxicology, Hammersmith Campus, Imperial College London, Du Cane Road, London, W12 0NN UK; School of Life and Medical Sciences, University of Hertfordshire, Hatfield, AL10 9AB UK; Synthesis Section, Department of Chemistry, Imperial College London, South Kensington, London, SW7 2AZ UK; Center for Materials Science, Zewail City, Sheikh, Zayed District, 12588, 6th of October City, Giza, Egypt

**Keywords:** Pulmonary arterial hypertension, Nanomedicine, Nanoparticles, Nitric oxide, NO-releasing nanoparticles, Endothelial cells, Pulmonary artery vascular smooth muscle cells, Pulmonary artery, Aorta

## Abstract

Pulmonary arterial hypertension (PAH) is a chronic and progressive disease which continues to carry an unacceptably high mortality and morbidity. The nitric oxide (NO) pathway has been implicated in the pathophysiology and progression of the disease. Its extremely short half-life and systemic effects have hampered the clinical use of NO in PAH. In an attempt to circumvent these major limitations, we have developed a new NO-nanomedicine formulation. The formulation was based on hydrogel-like polymeric composite NO-releasing nanoparticles (NO-RP). The kinetics of NO release from the NO-RP showed a peak at about 120 min followed by a sustained release for over 8 h. The NO-RP did not affect the viability or inflammation responses of endothelial cells. The NO-RP produced concentration-dependent relaxations of pulmonary arteries in mice with PAH induced by hypoxia. In conclusion, NO-RP drugs could considerably enhance the therapeutic potential of NO therapy for PAH.

Pulmonary arterial hypertension (PAH) continues to carry very poor prognosis in terms of both survival and quality of life in spite of the use of modern combination therapy [1]. This could be, at least in part, due to the fact that the exact molecular mechanisms remain largely unknown. The pathophysiology of the disease includes pulmonary vasospasm, vascular remodeling, and right ventricular failure. Several pathways have been implicated in the pathophysiology of PAH [2]. This has stimulated development of agents that can influence these pathways. One of the most promising strategies is modulating the nitric oxide (NO) pathway due to the apparent central role of NO in this disease [3]. NO is a potent vasodilator with anti-proliferative and anti-coagulant effects [3]. Several attempts at using inhaled NO gas in humans with both acute and chronic PAH have been tried [4]. The effect of NO gas is grossly limited by its short half-life and metabolism of cGMP by phosphodiesterase (type I and V) enzymes. In addition, adverse effects limit the systemic use of NO donors. Some of these limitations could be overcome by inhalation therapy particularly if combined with the use of a slow release nanoparticle formulation. There are several types of particles that may be useful as carriers of NO for the treatment of PAH including polymeric carriers [5]. Polymers have the advantage as potential drug carriers because they are biocompatible and their surface can be readily modified [5].

In an attempt to circumvent these major limitations, we have developed and performed extensive preclinical testing of a new NO-nanomedicine formulation. The NO-releasing polymers (NO-RPs) were prepared via ionotropic gelation technique. In brief, acidic solution of methyl silicate (2.8 ml/ml) was sonicated in ice bath at a power of 45 kW with 10 s pulse on and 5 s pulse off, followed by adding 1.1 ml of oligochitosan solution (0.5 % *w*/*v*, 15 KDa), polyvinylpyrrolidone (6.25 mg, 40 kDa), and 2.1 ml of polyethylene glycol (0.4 kDa) with stirring for 5 min. Afterwards, 15 ml of nitrite solution (3 % *w*/*v*) containing a predetermined weight of reducing agent was added followed by dropwise addition of 5 ml aqueous sodium tripolyphosphate (0.08 % *w*/*v*) with stirring. The resulting mixture was left at ambient temperature for 30 min followed by freeze-drying. The NO-RPs were characterized using Fourier transform infrared, X-ray diffraction, differential scanning calorimetry, scanning electron microscopy (SEM) and high-resolution transmission electron microscopy (HR-TEM). The particle size, as determined by dynamic light scattering, was in the range of 200–230 nm. This size was also confirmed using SEM and HR-TEM, which demonstrated a fine structure of the NO-RPs particles with a non-spherical morphology (Fig. 1a).

**Fig. 1:**
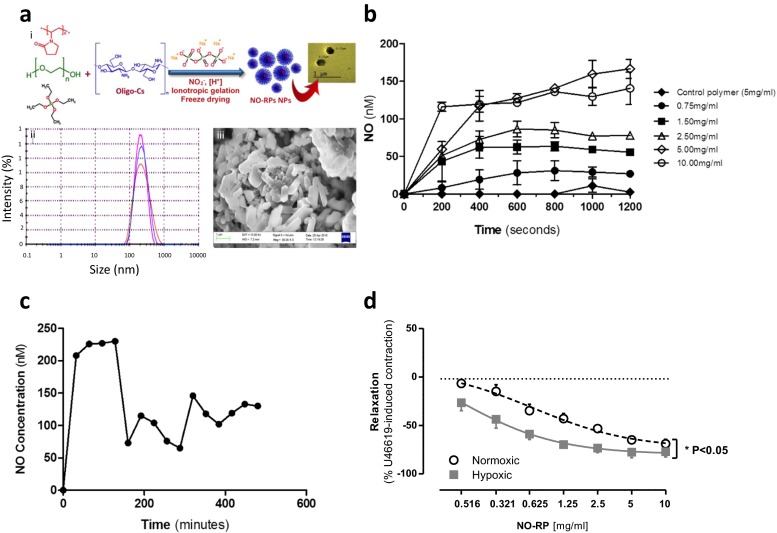
**a** (i) A schematic illustration of the development of the NO-releasing polymer and transmission electron microscopy image of the particles, size and morphology of the NO-releasing polymers as determined by (ii) dynamic light scattering and (iii) scanning electron microscopy at ×3035. **b** NO release from different concentrations of NO-releasing polymers (NO-RPs). Measurements were recorded using NO measuring electrode over a period of 20 min. **c** NO release pharmacokinetics from the NO-releasing polymers (NO-RPs). Measurements were recorded using NO measuring electrode over a period of 8 h. **d** Effect of NO-releasing polymer (NO-RP) on pre-contracted pulmonary artery from control mice and mice with pulmonary arterial hypertension. Data represents mean ± SEM for *n* = 3 from three animals. Statistical significance was determined by two-way ANOVA followed by a Bonferroni post hoc test (*p < 0.05)

In aqueous solution, simulating in vivo conditions, the NO-RP released NO in a concentration dependent manner with steady-state kinetics noted at 6.6 min (Fig. 1b) compared to NO release by SNP which was 6-times higher than the NO-RP at the same time point. When measured up to 8 h, NO release from the NO-RPs at a concentration of 5 mg/ml revealed two phases with an early peak lasting approximately 120 min followed by a decline (approximately 50 %) and a new steady state lasting for at least 8 h (Fig. 1c). When hemoglobin was added to the reaction mixture containing either NPs or SNP, NO release (measured by an NO-electrode) was quenched, confirming that free NO, rather than a higher oxide, was being detected.

At concentrations up 10mg/ml both control and the NO-RPs showed no effect on viability of endothelial cells under control culture conditions or in experiments where LPS was added to induce inflammation. Similarly, NO-RPs had no effect on basal or LPS-induced CXCL8 release from endothelial cells. The NO-RPs demonstrated no effect on pulmonary artery smooth muscle cell viability.

NO-RPs induced concentration-dependent relaxations of both aorta and pulmonary arteries (Fig. 1d). It was interesting to note that the NO-RPs appeared selective for vessels from mice with pulmonary hypertension displaying an increased EC_50_ compared to pulmonary arteries from control mice. SNP similarly induced vasodilator responses in pulmonary artery from both control mice and mice with pulmonary hypertension.

The current findings are essentially preliminary in nature and these findings need to be investigated further with additional experiments. In vivo studies with NO-RPs are required as there is a need to establish a clear benefit over existing therapeutic strategies in use of these molecules in the treatment of PAH.

These observations do show that NO-RPs (i) release free NO in aqueous solution, (ii) are non-toxic to cells and (iii) relax systemic (aorta) and pulmonary vessels. Our findings that this novel class of molecules appeared to selectively target vessels from mice with pulmonary hypertension is interesting and supports the idea that these preparations may be useful in the treatment of human disease.
